# Plant chemical genetics reveals colistin sulphate as a SA and NPR1-independent *PR1* inducer functioning via a p38-like kinase pathway

**DOI:** 10.1038/s41598-019-47526-5

**Published:** 2019-08-01

**Authors:** Vivek Halder, Mohamed N. S. Suliman, Farnusch Kaschani, Markus Kaiser

**Affiliations:** 1 Chemical Biology Laboratory, Max Planck Institute of Plant Breeding Research, Carl-von-Linnè-Weg 10, 50829 Köln, Germany; 2 Chemical Biology, Centre of Medical Biotechnology, Faculty of Biology, University of Duisburg-Essen, Universitätsstr. 2, 45141 Essen, Germany; 3 Present Address: Rijk Zwaan, De Lier, 2678 ZG The Netherlands; 4 Present Address: Desert Research Centre, 11753 El matareya, Cairo, Egypt

**Keywords:** Chemical genetics, Mechanism of action

## Abstract

In plants, low-dose of exogenous bacterial cyclic lipopeptides (CLPs) trigger transient membrane changes leading to activation of early and late defence responses. Here, a forward chemical genetics approach identifies colistin sulphate (**CS**) CLP as a novel plant defence inducer. **CS** uniquely triggers activation of the *PATHOGENESIS-RELATED 1* (*PR1*) gene and resistance against *Pseudomonas syringae* pv. *tomato* DC3000 (Pst DC3000) in *Arabidopsis thaliana* (Arabidopsis) independently of the *PR1* classical inducer, salicylic acid (SA) and the key SA-signalling protein, NON-EXPRESSOR OF PR1 (NPR1). Low bioactive concentration of **CS** does not trigger activation of early defence markers such as reactive oxygen species (ROS) and mitogen activated protein kinase (MAPK). However, it strongly suppresses primary root length elongation. Structure activity relationship (SAR) assays and mode-of-action (MoA) studies show the acyl chain and activation of a ∼46 kDa p38-like kinase pathway to be crucial for **CS’** bioactivity. Selective pharmacological inhibition of the active p38-like kinase pathway by SB203580 reverses **CS’** effects on *PR1* activation and root length suppression. Our results with **CS** as a chemical probe highlight the existence of a novel SA- and NPR1-independent branch of *PR1* activation functioning via a membrane-sensitive p38-like kinase pathway.

## Introduction

In nature, plants are in a constant exposure to a plethora of harmful and beneficial microorganisms. Recognition of pathogenic molecular cues such as microbe-associated molecular patterns (MAMPs) or effector molecules results in MAMP-triggered immunity (MTI) or effector-triggered immunity (ETI), respectively^1^. These recognition events initiate a variety of early defence responses such as ROS production, MAPK activation as well as triggers downstream signalling mediated via phytohormones such as SA, jasmonic acid, and ethylene^2^. The small phenolic phytohormone SA plays a critical role in plant defence by inducing systemic acquired resistance (SAR)^3^. Pathogen attack triggers the expression of SA-biosynthetic gene *ISOCHORISMATE SYNTHASE1* (*ICS1*)^4^ leading to an accumulation of SA which in turn is regulated by key regulators such as EDS1^5^. SA is perceived by two proteins, NPR1^6^ as well as the two NPR1 paralogs, NPR3 and NPR4^7^. Increase in SA-levels monomerises NPR1 causing its translocation to the nucleus and subsequent expression of defence genes such as *PR1*.

An ability to modulate plant defence signalling processes in a spatio-temporal manner offers several advantages for crop protection^8^. Chemical genetics i.e. use of small bioactive chemicals as probes has now become an established approach to study plant biological responses. It has been applied to study a variety of phytohormones signalling networks, endomembrane trafficking as well as cell wall formation^9,10^. Chemical screenings have been instrumental in identifying SA-like defence inducing compounds^11^. Some well-characterised examples include: benzothiadiazole (BTH)^12^, 2,6-dichloro-isonicotinic acid (INA)^13^, 3,5-dichloroanthranilic acid (DCA)^14^, probenazole^15^, imprimatins^16^ etc., amongst others. All these chemicals induce canonical SA marker genes such as *PR1* and require key SA-signalling components or SA itself for their bioactivity^11^.

In recent years, a number of CLPs such as surfactin, iturin, and fengycin etc., secreted by plant growth promoting rhizobacteria (PGPR), have been investigated for their defence-priming properties in plants. CLPs are natural products with wide structural diversity and strong antibiotic properties. They are synthesised by bacterial non-ribosomal peptide synthetases (NRPS) and share an overall similar architecture characterised by hydrophobic and acidic amino acids in their ring structure coupled to a fatty acid chain of variable length^17^. These compounds are now considered as a new class of MAMPs^18–21^ and are strong candidates for both biological pesticides and defence inducers^22^. Bioactive CLPs at low concentrations can sensitise plant lipid membranes leading to onset of early and late defence responses^18,20,23^. However, knowledge about associated signalling components and pathways is still in its infancy.

The polymyxin class of CLPs are strong antibiotics secreted by *Paenibacillus polymyxa*^24^. Typically, all polymyxins consist of a heptacyclopeptide fragment attached to a linear tripeptide with a fatty acid chain of varying length at the N-terminus and are polycationic via incorporation of the non-canonical diaminobutryic acid (Dab)^25^. Polymyxin E (PmE) or **CS** is an FDA approved commercial antibiotic used against Gram-negative bacteria infections^26^. At high concentrations **CS** has a strong biosurfactant property; however, at low concentrations it can modulate a variety of other functions such as inhibiting prokaryotic Hsp90^27^ and eukaryotic Hsp70^28^ chaperone activities or inducing innate immunity in nematodes by targeting a p38 kinase^29^.

The p38 kinase is a well-established membrane osmosensor^30^ known to play key roles during oxidative stresses in animals^31^. Plants lack classical p38 kinases^32^; however, several plant studies have implicated the activation of a p38-like kinase pathway during osmotic stress^33,34^, ABA-induced stomatal closure^35^, and cellular redox flux^36^. **CS** has been found to activate p38 kinase pathway in animal systems^29,37^, but its impact on plant p38-like kinase pathway is so far unknown.

In this study, we report on **CS** as a novel plant defence inducer. We identified **CS** through a phenotypic forward chemical genetic screen using *PR1p::GUS* defence reporter plants and a library of well-annotated commercial drugs of the Prestwick library. **CS** uniquely induces the SA-marker gene *PR1* independently of both SA and NPR1 demonstrating its distinctness from many natural and synthetic plant defence inducers. Mode-of-action studies reveal **CS** does not require kinases involved in ROS or MAPK signalling rather, but works via an active 46 kDa p38-like kinase pathway.

## Results

### CS is a novel activator of the *PR1* defence gene in Arabidopsis

**Figure 1:**
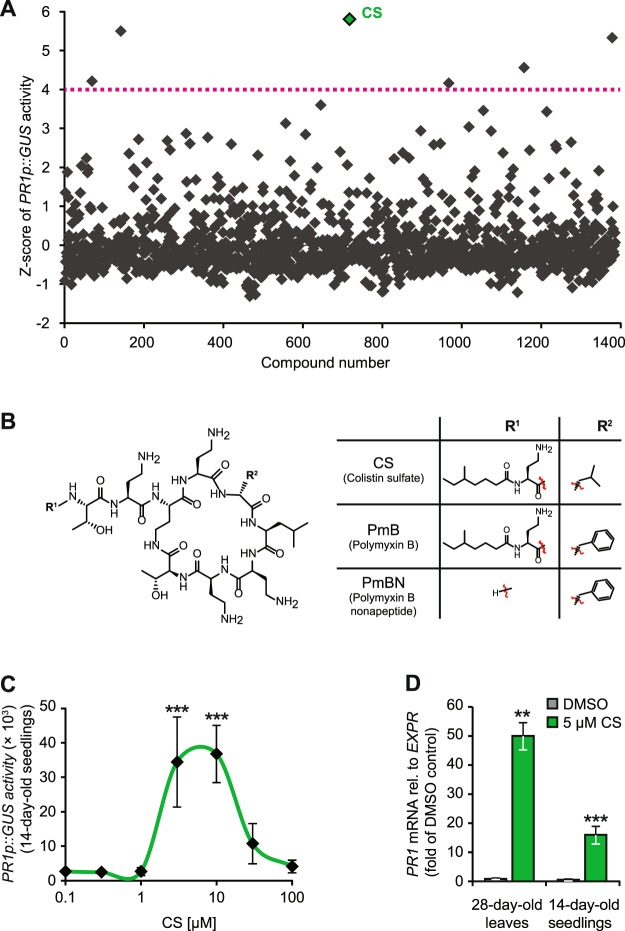
**CS** is a novel activator of the *PR1* defence gene in Arabidopsis. (**A**) Hydroponically grown (96-well microtiter plate format) 14-day-old *PR1p::GUS* Arabidopsis reporter seedlings were treated in duplicate with the compounds from the chemical library (20 µM) for 24 h. GUS activity was recorded and converted to Z-score values. Compounds above a Z-score of 4 (indicated by a magenta dotted line) were re-screened leading to the identification of **CS** (green diamond). **(B)** Chemical structure of **CS** and two related polymyxins: PmB and PmBN. **(C)** 14-day-old hydroponically grown *PR1p::GUS* reporter seedlings were treated with increasing concentrations of **CS** for 24 h before measuring GUS activity. (**D**) Leaves of 28-day-old and 14-day-old Col-0 plants were treated with DMSO (1%) or **CS** (5 µM) for 24 h. *PR1* transcript was quantified by qRT-PCR and normalised to the reference gene At4g26410 (Expressed Protein, *EXPR*). The values represent the mean (±SD) of at least three biological replicates. All experiments were repeated at least twice with similar outputs. Asterisks indicate significant differences from control values (*P < 0.05, **P < 0.01, and ***P < 0.001, two-tailed Student’s t-test).

The *PR1* gene is a well-known defence marker strongly responsive to SA accumulation upon pathogen attack^13^. Accordingly, monitoring *PR1* upregulation is an effective approach to identify potential plant defence activators^11^. Therefore, as a starting point to identify functional analogues of SA, we performed an *in planta* forward chemical genetic screen using the PCL (http://www.prestwickchemical.com/index.html) of 1280 small molecules (mostly approved drugs) along with 128 other handpicked compounds. We tested, in duplicates, 20 µM of each chemical on 14-day-old *PR1p::GUS*^38^ seedlings grown hydroponically in a 96-well plate format under long day conditions. Incubation with 200 µM SA or the solvent DMSO (1%) served as internal controls and bioactivity of compounds on *PR1p::GUS* induction was scored via an *in situ* GUS assay^39^. The resulting GUS-activity was converted to Z-scores as a criterion for hit-selection (Fig. 1A). For a confident primary-hit selection, we set a stringent threshold of Z-score ≥ 4 to focus on the strongest candidates with a bioactivity at least 4 times or higher than the DMSO control. In total, we identified six primary hits: acetyl salicylic acid (present twice in the chemical collection), 4-methyl umbelliferone, alexidine dihydrochloride, isradipine, and **CS**. About 80% of all tested compounds displayed a Z-score of around zero, indicating no effect on *PR1* induction.

All six primary hits were retained in the secondary screenings. However, we decided to proceed further with **CS** as (i) **CS** and other polymyxins such as polymyxin B (PmB) and polymyxin B nonapeptide (PmBN)^40^ were commercially available (Fig. 1B), (ii) acetyl salicylic acid is a known SA-derivative^41^ and 4-methyl umbelliferone corresponded to the *in situ* GUS assay readout^39^, and therefore might well be an artefact (iii) **CS** displayed stronger bioactivity than alexidine dihydrochloride and isradipine, and (iv) unlike the other well-studied surfactin-type CLPs, **CS** polymyxins were never investigated in Arabidopsis before. To confirm the observed *in planta* effect of **CS**, we first performed a dose-response assay using 0.1 to 100 µM **CS** (Fig. 1C). *PR1p::GUS* expression steadily increased in a dose-dependent manner until 10 µM and declined sharply at ≥30 µM indicating the onset of toxic effects. The resulting half maximal effective concentration (EC_50_) of **CS** for *PR1* induction was 5 µM. Next, we validated **CS’** bioactivity, *in vivo*, by quantifying its effect on the accumulation of endogenous *PR1* mRNA levels. Hydroponically grown 14-day-old Col-0 seedlings and soil-grown 28-day-old mature Col-0 plants were treated with 5 µM **CS** for 24 h. In both cases, **CS** strongly triggered a robust increase of *PR1* transcripts compared to the DMSO controls (Fig. 1D). These results confirmed **CS**-mediated *PR1* induction and also the robustness of our *in situ* GUS assays as a tool for chemical screenings. *PR1p::GUS* induction was detectable already after 30 min and steadily increased up to 24 h (Supplemental Fig. S1). Altogether, our results indicate that **CS** is a novel exogenous activator of the defence gene *PR1* in Arabidopsis.

### CS does not stress plants at its EC_50_ but affects seed germination and root growth

**Figure 2:**
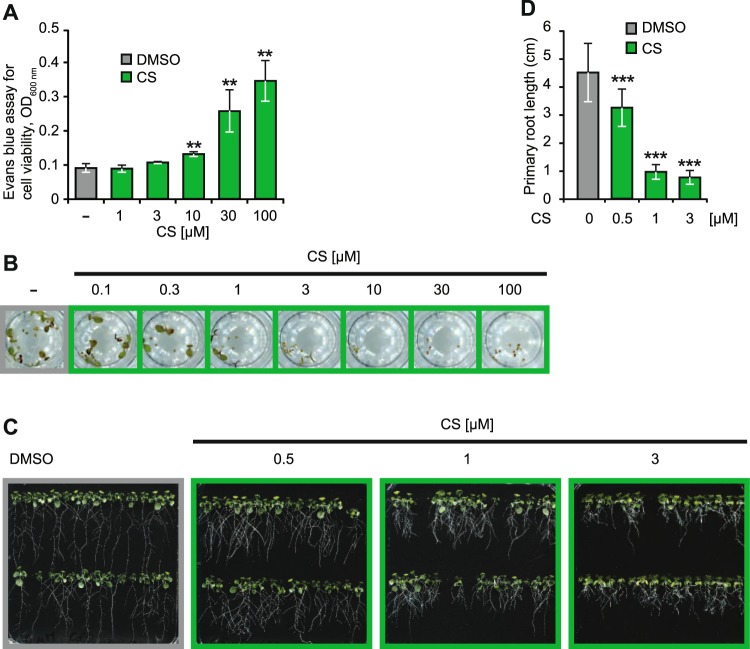
**CS** does not stress plants at its EC_50_ but affects seed germination and root growth. **(A)** 14-day-old Col-0 seedlings, treated with increasing concentrations of **CS** for 24 h were vacuum-infiltrated with Evans blue (0.1%, w/v) for 30 min and then kept for at least 6 h in the vacuum chamber. Dye bound to dead cells was quantified at A_600_/A_680_. **(B)** Properly sterilised and stratified Col-0 seeds were hydroponically grown for a week with the indicated **CS** concentration under long day conditions. **(C)** Properly sterilised Col-0 seeds were sown on solid half-MS phytagel media plates containing indicated concentrations of **CS**. Seeds on plates were stratified for 48 h and then kept vertically for 14 days in a long day growth chamber. **(D)** Root length of plants in **(C)** were quantified using the ImageJ software. At least 10 to 15 seedlings were used for root length calculation. All experiments were repeated at least twice with similar outputs. Asterisks indicate significant differences from respective controls (**P < 0.01 and ***P < 0.001, two-tailed Student’s t-test).

**CS** is a well-known biosurfactant courtesy of its cationic L-diamino-butyric acids (L-Dab) that interact with the anionic lipopolysaccharide molecules of Gram-negative bacteria to induce membrane disruption^40^. Plant membranes; however, have a different chemical composition than Gram-negative bacteria and hence certain CLPs such as surfactin are able to interact with plant membranes without inducing concomitant damage at low concentrations^20,23^. To test whether **CS** displays similar properties, we incubated Arabidopsis seedlings for 24 h with increasing concentrations of **CS**, followed by a subsequent Evans blue-based cell death quantification assay. In line with earlier reports for surfactin-type CLPs^20,23^, we observed cell toxicity only at ≥10 µM, indicating lower concentration of 5 µM are well-tolerated in two-week-old seedlings (Fig. 2A). Next, to understand the long-term effect of **CS** on plant growth and development, we germinated Col-0 seeds hydroponically in presence of 0 to 100 µM **CS**. A marked reduction in seed germination was noticed starting at 1 µM with complete inhibition at ≥3 µM (Fig. 2B). The seed germination assays showed a marked impact of **CS** on root emergence starting at concentrations of 1 µM. Upon further examination, we found that 0, 0.1, 0.5, and 3 µM **CS** impart a dose-dependent inhibitory effect on primary root growth elongation (Fig. 2C,D). Noticeably, a clear increase in root branching was observed upon application of **CS** (Fig. 2C), which strongly resemble the impact of its producer *Paenibacillus polymyxa* on its host root phenotype^42^. Furthermore, the impact of **CS** on root growth was independent of auxin signalling (Supplemental Fig. S2). Overall, our results indicate that **CS** triggers plant immunity along with an impact on plant root growth.

### CS’ unique MoA induces *PR1* independent of SA and SA-signalling components

**Figure 3:**
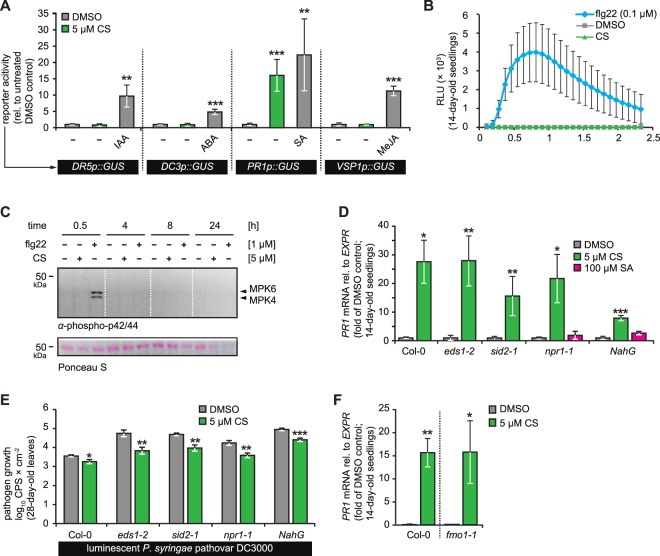
**CS’** unique MoA induces *PR1* independent of SA and SA-signalling components. (**A**) 14-day-old GUS reporter seedlings, inducible by selective phytohormones, were treated either with **CS** (5 µM) or with the respective inducers IAA (5 µM), ABA (100 µM) or MeJA (100 µM)) for 24 h in at least 3 biological replicates. GUS activity was normalised to the respective DMSO control of each reporter line. **(B)** 14-day-old Col-0 seedlings were treated with DMSO (1%), **CS** (5 µM), and flg22 (0.1 µM) followed by immediate addition of luminol-peroxidase mix to detect chemiluminescence [(reported as relative light units (RLU)] by a luminometer for the indicated time course. The values represent the mean (±SD.) of at least 5–10 biological replicates. **(C)** Hydroponically grown Col-0 seedlings were treated with bacterial elicitor Flg22 (1 µM), **CS** (5 µM) and DMSO (1%) for indicated time durations before harvesting and detecting phosphorylated MPK6 and MPK3 bands by immunoblotting using α-phospho-p44/42. For the uncropped blot, see Supplemental Fig. S7. **(D)** Hydroponically grown, 14-day-old seedlings of indicated genotypes were treated with DMSO (1%), **CS** (5 µM) or SA (100 µM) for 24 h. *PR1* gene levels were plotted as change fold to the respective DMSO controls of each genotype. The values represent the mean (±SD) of at least three biological replicates. **(E)** Four-week-old leaves of different Arabidopsis genotypes were infiltrated with DMSO (1%) or **CS** (5 µM) for 24 h before spray-inoculating with a virulent luminescent luxCDABE-tagged Pst DC3000 strain (Fan *et al*., 2008). Pathogen growth was plotted as log_10_ CPS per cm^2^ relative to the DMSO control of each genotype. **(F)** 14-day-old Col-0 wild-type and *fmo1-1* mutant seedlings were treated with DMSO (1%) and **CS** (5 µM) for 24 h. *PR1* transcripts were quantified via qRT-PCR and are reported relative to *EXPR*. The values represent the mean (±SD) of at least three biological replicates. All experiments were repeated at least twice with similar outputs. Asterisks indicate significant differences from respective DMSO controls (*P < 0.05, **P < 0.01, and ***P < 0.001, two-tailed Student’s t-test).

Both natural and synthetic CLPs can trigger early or late defence responses such as ROS production and/or both SA and JA activation, respectively^18–20,23,43–46^. For compounds of the polymyxin class, in particular **CS**, we anticipated similar responses and, therefore, first tested its effect on three major phytohormonal signalling pathways: (i) JA-responsive *VSP1p::GUS*^47^, (ii) Auxin responsive *DR5p::GUS*^48^, and (iii) ABA responsive *DC3p::GUS*^49^. **CS** did not induce the reporter lines of JA, Auxin, and ABA, but SA-signalling thereby indicating its selectivity towards *PR1* defence gene activation (Fig. 3A). Next, we tested effect of **CS** on ROS production, which is an excellent marker of stress induction upon CLPs application and is also known to trigger SA signalling. Col-0 seedlings treated with 5 µM **CS** showed no induction of ROS production while flg22, the classical ROS inducer, caused a clear ROS burst (Fig. 3B). Nevertheless, to eliminate the possibility of a late ROS induction contributing to **CS’** bioactivity, we tested it on the ROS deficient *rbohd/f* mutant^50^. Again, **CS** caused a strong induction of *PR1* in both wild-type as well as the *rbohd/f* mutant (Supplemental Fig. S3). Similarly, another defence regulator, the MAPKs (MPK6 and MPK3)^51^, were not induced by **CS** and no transient activation was noticed (Supplemental Fig. S4). Moreover, **CS** did not trigger sustained MAPK activation, a known pathway for SA-independent *PR1* activation^51^, at later time points of 4, 8, and 24 h, while flg22 triggered a transient MAPK activation (Fig. 3C). Our results suggest that **CS**-induced signalling works via a unique pathway independent of ROS and MAPK components.

*PR1* is a canonical marker gene of SA and its expression is severely impaired in SA-signalling mutants^52^. To investigate whether **CS** requires SA or its associated components in Arabidopsis, we tested its response in mutants of (i) SA biosynthesis: *sid2-1*^4^, (ii) SA-accumulation: *NahG* plants^52^, (iii) SA signalling regulation: *eds1-2*^5^, and (iv) SA signalling transduction: *npr1-1*^53^. Surprisingly, application of 5 µM **CS** induced *PR1* expression in all genotypes whereas SA as expected failed to induce *PR1* expression in *npr1-1*^54^ and *NahG* plants^55^ (Fig. 3D). These results, therefore, indicated a unique bioactivity of **CS** different from several well characterised *PR1* inducers^11^. Similar, albeit a slightly varying, bioactivity was observed in 28-days-old plants of same genotypes (Supplemental Fig. S5). We next tested whether **CS** induces plants’ resistance to bacterial infection. We syringe-infiltrated leaves of 28-day-old wild-type as well as the SA-signalling and SA-deficient plants with or without 5 μM **CS** for 24 h, before injecting the leaves with a luminescent luxCDABE-tagged Pst DC3000 strain^56^ for rapid and reliable quantification of bacterial growth. In accordance with the results from CS-triggered *PR1* gene expression induction (Fig. 3D), **CS** application enhanced resistance in all tested Arabidopsis mutant genotypes (Fig. 3E).

We next sought out to find whether **CS** works via the SA-independent signalling branch. We tested the well-characterised *fmo1-1* mutant defective in the FLAVIN-DEPENDENT MONOOXYGENASE1 (FMO1) protein regulating SA-independent resistance^57^. Surprisingly, **CS** triggered strong *PR1* expression in *fmo1-1* plants (Fig. 3F) akin to wild-type. In summary, our findings indicate **CS**-triggered *PR1* induction to be independent of both SA-dependent and SA-independent signalling factors and therefore occurs via an unconventional pathway.

### Structure-activity-relationship assays with CS

**Figure 4:**
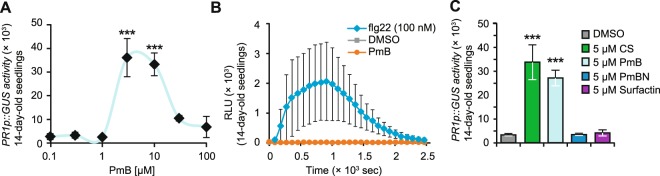
Structure-activity-relationship assays with **CS**. **(A)** 14-day-old hydroponically grown *PR1p::GUS* reporter seedlings were treated with increasing concentrations of PmB for 24 h before measuring GUS activity. **(B)** 14-day-old Col-0 seedlings were treated with DMSO (1%), PmB (5 µM), and flg22 (0.1 µM) followed by immediate addition of luminol-peroxidase mix to detect chemiluminescence (reported as relative light units (RLU)) by a luminometer for the indicated time course. The values represent the mean (±SD) of at least 5–10 biological replicates. **(C)** 14-day-old *PR1p::GUS* seedlings were treated with **CS** (5 µM), PmB (5 µM), PmBN (5 µM) and surfactin (5 µM) for 24 h before quantifying GUS activity. The values represent the mean (±SD) of at least 3 biological replicates. All experiments were repeated at least twice with similar outputs. Asterisks indicate significant differences from respective controls (***P < 0.001, two-tailed Student’s t-test).

To better understand the MoA of **CS** and its essential structural constituents, we tested some commercially available **CS** derivatives^40^. First, we tested PmB, an analogue with a d-phenyl alanine instead of d-leucine at ring position 6 of **CS** (Fig. 1A). Dose-response assays with PmB on *PR1p::GUS* displayed almost identical EC_50_ values of ca. 5 µM. Also, as observed with **CS**, PmB strongly reduced plant fitness at 30 µM (Fig. 4A) and failed to induce ROS production (Fig. 4B). We thereby concluded that the close structural homolog PmB displays **CS** like bioactivities. Next, we focused on the two structural ‘regions’ of polymyxins critical for their bioactivity: the fatty acid chain^58^ and the pentacationic polypeptide fragment^59^. We tested Polymyxin B nonapeptide (PmBN), an enzymatically derived PmB derivative lacking the fatty acid chain^60^ and surfactin a well characterised CLP with different amino acid composition from **CS**^18,23,44^ (Fig. 1B). While **CS** and PmB strongly induced *PR1p::GUS* expression, 5 µM of both PmBN and surfactin showed no agonistic effects (Fig. 4C). These results indicate that **CS** and PmB display a structure specific mode-of-action and the fatty acid chain is indispensable for its bioactivity.

### CS functions via a stress-sensitive p38-like protein kinase pathway

The PCL is comprised of well-annotated compounds used already in different model systems^61–63^ but plants. We reasoned the knowledge gained from previous PCL-based screens, and specifically with **CS**, could therefore be useful to unravel the molecular mechanisms of **CS** in plants. Notably, from a recent chemical genetic study with PCL on living *C. elegans*, **CS** was identified as a defence inducer functioning via an active p38 kinase signalling pathway^29^. We thus hypothesized **CS** might have similar mode-of-action in plants since the p38-like kinase pathway is known to exist in Arabidopsis^35,36,64^, wheat root cells^65^, and sweet potato^66^ in addition to other species such as algae^67^.

**Figure 5:**
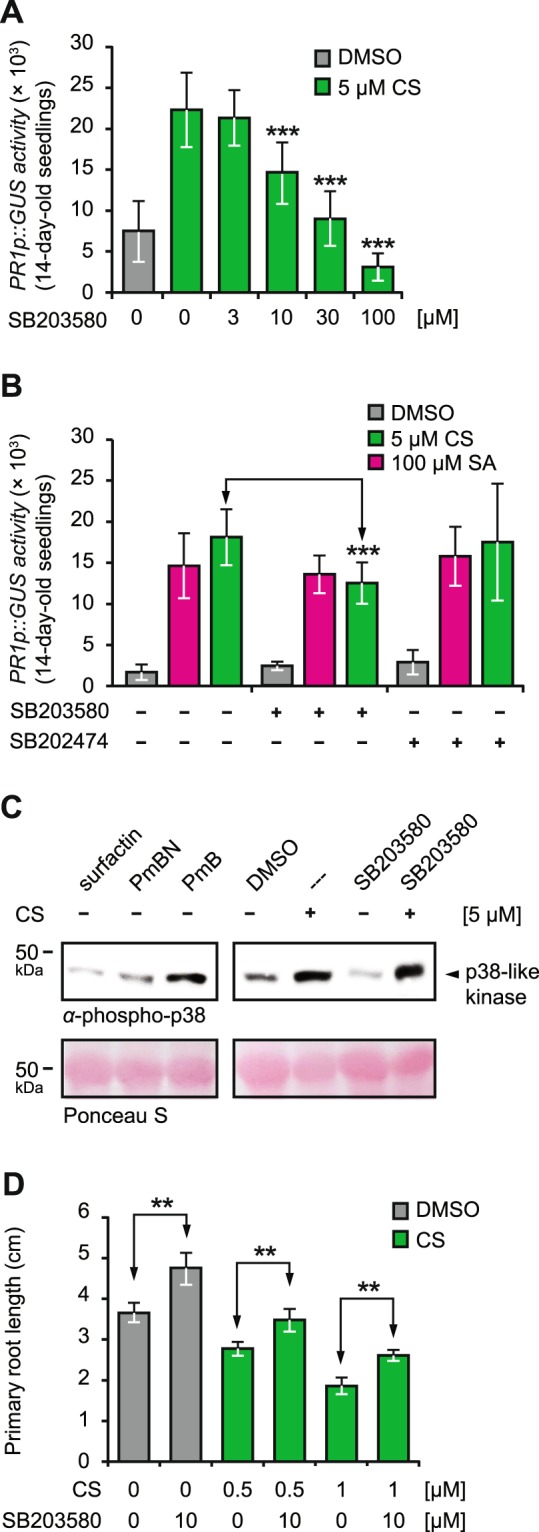
**CS** functions via a stress-sensitive p38-like protein kinase pathway. (**A**) 14-day-old *PR1p::GUS* reporter seedlings were pre-treated for 1 h with increasing concentrations of SB203580, followed by application of **CS** (5 µM) for 24 h before measuring resulting GUS activity. **(B)** 14-day-old *PR1p::GUS* reporter seedlings were pre-treated for 1 h with either SB203580 (10 µM) or SB202474 (10 µM) and were supplemented with **CS** (5 µM) and SA (100 µM) as indicated. **(C)** Western blot analysis of protein extracts from 14-day-old Col-0 wild-type seedlings treated with surfactin (5 µM), PmBN (5 µM), PmB (5 µM), **CS** (5 µM), or DMSO (1%) and the p38-like kinases inhibitor SB203580 (10 µM) either alone or together with **CS** (5 µM). Total protein was extracted and after gel electrophoresis and western blotting the membrane was probed with a commercially available α-phospho p38 antibody. Equal protein loading is demonstrated by Ponceau S staining. For the uncropped blot, see Supplemental Fig. S8. **(D)** Properly sterilised Col-0 seeds were sown on solid half-MS phytagel media plates containing indicated concentrations of **CS** and 10 µM SB203580. Root length were quantified using the ImageJ software. All experiments were repeated at least twice with similar outputs. Asterisks indicate significant differences from respective controls (***P < 0.001, **P < 0.01, two-tailed Student’s t-test).

We began with a highly selective mammalian p38-kinase pathway inhibitor, SB203580^68^, used frequently in plant studies^35,36,65,66^, to see whether it ablates **CS’** bioactivity? 14-day-old *PR1p::GUS* seedlings were pre-treated with 3–100 µM SB203580 1 h before supplementing them with 5 µM **CS** for 24 h. Interestingly, application of SB203580 at ≥10 µM significantly diminished **CS’** bioactivity (Fig. 5A). To avoid off-target effects at higher concentrations, we decided to use 10 µM SB203580 for all future experiments. To confirm the observed reversal of **CS’** bioactivity by SB203580 is due to the interference of p38-like kinase pathway and not due to any off-target effect, we tested SB202474, an inactive SB203580 analogue having negligible effect on mammalian p38 kinase signalling^69^. SB202474 failed to suppress **CS** triggered *PR1p::GUS* activation indicating that the p38-like kinase pathway is indeed involved in *PR1* activation by **CS**. Finally, to test whether SA also requires the p38-like kinase, we tested 10 µM of both SB203580 and SB202474 on SA treated *PR1p::GUS* seedlings. SB202474 showed no effect, but more remarkably SB203580 failed to reverse SA-triggered *PR1* activation (Fig. 5B).

The p38 kinase in a well-known membrane sensor shown to be rapidly phosphorylated upon application of **CS** in *C.elegans*^29^ and mice^37^. We, therefore, asked whether **CS** phosphorylates (i.e. activate) the p38-like kinase pathway in Arabidopsis? Taking advantage from our previous SAR studies, we treated 14-day-old Col-0 seedlings with 5 µM of **CS**, PmB, PmBN, and surfactin for 24 h before detecting the phosphorylation status via Western blots. Only **CS** and its close structural and functional analogue PmB, induced the phosphorylation of a protein in ∼46 kDa detected by anti-phospho-p38 antibody (Fig. 5C). This protein band and antibody has been identified and used, respectively, in a number of reported studies^36,65–67,70,71^. Furthermore, in line with our previous findings in Fig. 4C, the functionally inactive **CS** derivative, PmBN, as well as the structurally divergent CLP, surfactin, did not trigger any phosphorylation (Fig. 5C). The inability of SB203580 to affect **CS** triggered phosphorylation was in accordance with previous reports demonstrating that SB203580 acts downstream of an activated p38 kinase pathway^68^. Collectively, our results present **CS** polymyxins as novel exogenous *PR1* inducers in Arabidopsis working via an active p38-like kinase pathway.

Finally, application of SB203580 also reversed **CS** mediated primary root growth suppression (Fig. 5D), indicating it works via the p38-like kinase. Moreover, application of **CS** to tomato (*Solanum lycopersicum* cv. Moneymaker) leaf discs triggered significant *PR1* upregulation, likewise Arabidopsis, signifying it may have useful application in other plants (Supplemental Fig. S6).

## Discussion

Using our well established *in planta* forward chemical genetic screening on whole Arabidopsis *PR1p::GUS* reporter seedlings^39^, we identified two polymyxins, **CS** and PmB, as a novel class of exogenous plant defence modulators and strong inducers of the canonical SA-marker gene, *PR1*. Our mode-of-action experiments revealed that **CS** requires an active p38-like kinase signalling for its bioactivity. **CS** triggers the phosphorylation of a 46 kDa protein that shares strong biochemical and immunological similarity to the mammalian p38 kinase. Strikingly, **CS**-triggered *PR1* expression was independent of SA as well as classical SA-dependent and SA-independent signalling components. This is unlike many of the established exogenous SA functional mimics^11^ and CLPs^21^.

**CS** belongs to the polymyxin family of CLPs. These compounds are biosynthesised by PGPRs found as beneficial root dwellers in host plants^72^. The plant root microbiome, including many CLP producing PGPRs, has recently been recognised as a novel modulator of plant innate immunity^73^. Functionally, **CS** is a potent biosurfactant used as an antibacterial drug^26,74^. Besides its well-known antibacterial properties, **CS** was recently identified as an exogenous modulator of animal innate immunity^29^. Nevertheless, the plant immune-modulatory properties of **CS**, and in general polymyxins, were unknown before our study. This is in a stark contrast to the rich body of literature on the immunomodulatory roles of other well-characterized CLPs such as surfactin in Arabidopsis^20,21,44^. Our findings demonstrate that despite both CLP families enhance plant immunity they nevertheless feature different mode-of-actions. In Arabidopsis**, CS** treatment resulted in a strong upregulation of endogenous *PR1* mRNA levels in both seedlings and mature plants. Furthermore, upregulation of *PR1* expression took place in a dose-dependent manner with strong toxic effects ensuing at 30 µM or higher. This was in accordance with the cell death assays which demonstrated **CS**-triggered toxicity at similar concentrations. Therefore, the cell death phenotypes at higher concentrations are most probably caused by the well-documented membrane disruption properties of **CS**. This effect was much pronounced in the seed germination and root growth assays where the plants were in constant exposure to **CS** for several days leading to the suppression of seed germination at ≥1 µM or primary root growth inhibition at ≥0.5 µM.

Plant membranes are key players for plant immunity as they mark the first line of defence and their integrity is therefore carefully monitored and a change in membrane fluidity can induce stress leading to defence induction^46,75^. Activation of ROS and MAPKs are some of the early responses upon perception of MAMPs and CLPs at plasma membrane. Surprisingly, **CS** did not require any of the signalling components involved in ROS and MAPK signalling many of which are located in the plasma membrane, making it a unique inducer of plant defence. However, it is clear CS trigger transient membrane changes to trigger downstream responses. This model is supported by our SAR studies which demonstrated PmB, a close functional analogue of **CS**, also generates strong *PR1* induction. In contrast, PmBN, a **CS** analogue without the membrane interacting lipid chain moiety and surfactin, a CLP with different amino acid and fatty acid chain length, failed to replicate **CS** like responses. These results suggest that membrane perturbation trigger is a result of some specific ‘electrostatic’ and lipid chain membrane interaction mediated by **CS** and the non-toxic concentration of 5 µM **CS** is too low to induce a complete collapse of membrane integrity but may cause membrane sensitisation. The low bioactive concentration of 5 µM **CS** in Arabidopsis is significantly lower than the high 50–100 µM bioactive concentration range of surfactin^76^ and iturin A^21^ in Arabidopsis and other plant species.

A unique feature which sets **CS** apart from existing chemical and natural plant defence inducers is its ability to induce defence in SA-deficient plants as well as mutants of SA-dependent and SA-independent signalling components. Classical synthetic SA-mimics such as BTH^77,78^, Probenazole^79^, INA^80^, and N-cyanomethyl-2-chloroisonicotinamide (NCI)^81^ etc., require either or both SA and NPR1. **CS**, however, can induce *PR1* expression in SA-deficient, *sid2-1* and *NahG*, plants as well as in the mutant of SA-signalling *npr1-1* and the mutant of SA-regulation *eds1-2*. CLPs such as iturin A of the surfactin family cannot upregulate *PR1* in an *npr1-5* mutant background^21^. Thus, our data suggest that **CS** displays a unique MoA. Furthermore, the ability of **CS** to trigger *PR1* induction can be seen translated in the Pst DC3000 infection assays where **CS** enhanced plant defence in all the tested SA and SA-signalling mutants. Since **CS** is a known biosurfactant, it is fair to assume it will have toxic effects on Pst DC3000 upon direct contact. In our studies we applied **CS** 24 h before pathogen application and also observed a varying growth of the bacteria across different mutant genotypes. Although we cannot completely rule out that the observed enhanced plant resistance vs. Pst DC3000 after **CS** treatment stems from its known antibiotic properties, the variance of bacterial growth in accordance with the mutant genotype indicates that **CS** also acts via modulation of plant defence signalling pathways.

The requirement of an active p38-like kinase pathway by **CS** to induce *PR1* expression is an intriguing discovery and is analogous to its bioactivity in *C. elegans*^29^. This also hints towards an evolutionary conserved role of this kinase in innate immunity. Even though, all known plant kinases lack the characteristic TGY (threonine-glycine-tyrosine) signature motif of animal p38 kinases^32^, many studies have strongly implicated the presence of a putative p38 homolog commonly called as “p38-like kinase”. Currently, no genetic mutants are available to test the significance of p38-like kinase in plant physiology. To overcome this bottleneck, plant biologists have created conditional knockdowns by using a highly selective mammalian p38 kinase pathway inhibitor, SB203580^68^, and have validated the outputs by using its inactive variant SB202474^35,36^. Similarly, scientists have routinely used the anti-phospho-p38-MAPK mammalian antibody to observe the activation of the p38-like kinase in plants such as sweet potato^66^, Arabidopsis^35,36^, perilla^82^, and wheat^65^ as well as in fungal species such as *Dunaliella viridis*^83^ or *Dunaliella tertiolecta*^70^, and algae^84^. The anti-phospho-p38-MAPK antibody does not cross-react with phoshpo-ERK proteins of plants^65^ underlying its specificity toward the p38-like kinase. We performed similar experiments using SB203580 and demonstrated that it severely hampers **CS**-triggered *PR1* induction. Remarkably, SB203580 did not inhibit SA-triggered *PR1* induction suggesting SA pathway works independent of p38-like kinase in Arabidopsis. Next, using the anti-phospho-p38-MAPK antibody, we showed that both **CS** and its analogue PmB can induce the phosphorylation of a 46 kDa protein kinase. Remarkably, surfactin, which is also a biosurfactant, and PmBN, an inactive **CS** derivative, could not trigger phosphorylation of the p38-like kinase. Another evidence of **CS** functioning via p38-like kinase was demonstrated by the root growth assay where SB203580 caused a significant reversion of **CS** induced root growth suppression. These results suggest a possible involvement of the p38-like kinase as a signalling juncture regulating growth and defence trade-off. However, further studies will be required to dissect this signalling branch. Nevertheless, our results suggest bioactivity of **CS** can well be a consequence of fine-tuned electrostatic interactions at the plant membrane or could also be transduced by a specific and yet unknown membrane-bound receptor leading to activation of the p38-like kinase pathway.

Overall, our results highlight an active p38-like kinase signalling pathway to be crucial for **CS** triggered *PR1* activation and possibly root growth suppression. We show that polymyxins such as **CS** and PmB can trigger the activation of a 46 kDa protein kinase pathway sharing significant biochemical and immunological similarity to mammalian p38 kinases. In future, compounds targeting p38-like kinase activity may find application as novel plant defence modulators.

## Materials and Methods

### Plant material

Transgenic *fmo1-1*^57^, *rbohd/f*^50^, *eds1-2*^5^, *npr1-1*^53^, *sid2-1*^4^, *NahG*^52^, *PR1p::GUS*^38^, *DC3p::GUS*^49^, *DR5p::GUS*^48^, and *VSP1p::GUS*^47^ in *Arabidopsis thaliana* (Col-0) background were used for various bioassays.

### Growth conditions

Surface-sterilised seeds were sown in 96-well plates (PerkinElmer Inc., Germany) containing half-MS basal salt (Sigma Aldrich) liquid medium supplemented with 0.5% sucrose. Seeds were stratified at 4 °C in the dark for 48 hours and later grown for 14 days under long day conditions (day/night cycle of 16/8 h at 21/19 °C). For soil grown Col-0, *sid2-1*, *npr1-1*, *eds1-2*, and *NahG* plants, seeds were sown directly on soil and stratified at 4 °C in the dark for 48 h. Plants were grown for about 28 days under long day conditions before performing biological assays.

### Chemicals

The tested chemicals were taken from the Prestwick Chemical Library (1280 compounds, Illkirch-Graffenstaden, France) in addition to a collection of 128 handpicked compounds. **CS** was purchased from AK Scientific (San Francisco, USA), surfactin and PmB were obtained from Santa Cruz Biotechnology (Dallas, USA), PmBN and SB203580 from Sigma (Deisenhofen, Germany), and SB202474 from Cayman chemicals (Michigan, United States).

### Activator screen using *PR1p::GUS* reporter

Screening was performed as described^39^. In brief, 14-day-old Arabidopsis seedlings harbouring the *PR1p::GUS* reporter were pre-treated with chemicals (dissolved in DMSO) at a final concentration of 20 µM for 24 h. Seedlings treated with 200 µM SA (dissolved in DMSO) or solvent DMSO (1%) served as internal controls. For each plant, at least two biological replicates were performed, and the resulting activity was normalised to control DMSO samples located on the same microplate.

### *in situ* GUS activity quantification

The assay was performed as described previously^39^. In brief, 14-day-old seedlings grown in 96-well microplates were submerged in 150 µL lysis buffer (50 mM sodium phosphate, pH 7.0, 10 mM EDTA, 0.1% Triton X-100), containing 1 mM 4-MUG, at 37 °C for 90 min. At the end of the incubation period, 50 µL 1 M Na_2_CO_3_ (stop solution) was added to each well and the resulting 4-MU fluorescence was directly determined in a microplate reader as described before (excitation/emission wavelength of 365/455 nm). Activity was directly expressed as relative light units (RLU per seedling).

### Root length quantification

Solid half-strength MS medium was supplemented with MES (0.5 g L^−1^), sucrose (5 g L^−1^), and 0.8% (w/v) Phytagel (Sigma Aldrich). Media was autoclaved and chemicals were added to media at 60 °C. Seeds were sown directly on the plates, stratified for 48 h, and grown for two-weeks under long day conditions. Images were taken and quantified using Image J software (https://imagej.nih.gov/ij/).

### Oxidative burst assay

Luminol-based oxidative burst measurement was performed with 10-day-old Col-0 seedlings in 96-well white microplates. Seedlings were submerged in 100 μL H_2_O supplemented with 5 µM **CS**, 1% DMSO or 0.1 µM flg22. Luminescence detection was started immediately in a luminometer (Centro LB960, Berthold Technologies, Germany) by adding 100 µM luminol (L-012 from Wako Chemicals, USA) and 10 µg mL^−1^ horseradish peroxidase (Sigma-Aldrich, P6782) to the wells. Luminescence was measured every minute for the next 30 minutes. At least 5–10 biological replicates were used for each measurement.

### Immunoblotting assays

Immunoblotting of MAPK as per^51^ and p38-like kinase as per^36^ was done with minor modifications. In brief, total protein was extracted from chemically-treated 14-day-old Arabidopsis Col-0 seedlings and 10 µg equal protein amounts were separated in 10% polyacrylamide gel by SDS-PAGE. For western blot, the gel was transferred to nitrocellulose membranes and probed with primary antibodies overnight at 4 °C. MAPK was detected using anti-phospho-p44/p42 MAPK antibodies (Cell Signalling Technology, 9102) while for p38-like kinase an anti-phospho p38-MAPK (Cell Signalling Technology, 9211) was used. Horseradish peroxidase-tagged goat anti-rabbit secondary antibodies were employed in both cases. Phosphorylation was detected using the Pierce ECL Western Blotting Substrate (Thermo Scientific, Rockford, USA) and imaged via ImageQuant LAS 4000 (GE Healthcare Life Sciences, Sweden).

### Cell death assay using Evans blue dye

Cell death was examined by Evans blue staining as per^85^ with minor modifications. Briefly, 14-day-old Col-0 seedlings were vacuum-infiltrated in Evans blue (0.1% w/v) for 30 min and then maintained for at least 6 h in the vacuum chamber. To remove unbound dye post staining, extensive washing with autoclaved water was done. Dye bound to dead cells was solubilised in methanol (50% v/v) and SDS (1%) for 30 min on a shaker and quantified in a spectrophotometer at A_600_/A_680_.

### Quantitative real-time PCR analysis

Total plant RNA was extracted using the manufacturer’s protocol (Bio-Budget technologies GmbH, Germany) and was used to make first strand cDNA using SuperScript™ II Reverse Transcriptase (Invitrogen). The cDNA was used as template in PCR reactions using gene specific primers (Sigma, Germany) and iQ™ SYBR® Green Supermix (Bio-Rad, Germany) on an iQ5 Real-Time PCR Detection System (Bio-Rad, Germany). The experiment was performed in three biological replicates (with one technical replicate for each biological replicate). The following primers (5′-3′) were used in our study:

### Primers for arabidopsis

At*EXPR* (reference gene) FP: GAGCTGAAGTGGCTTCCATGAC

At*EXPR* RP: GGTCCGACATACCCATGATCC

At*PR1* FP: TCACAACCAGGCACGAGGAG

At*PR1* RP: CACCGCTACCCCAGGCTAAG

### Primers for Tomato

*SlHKG4* (reference gene) FP: GCTCCAGAAAGCTACATC

*SlHKG4* RP: CGTCTCCTATAACGACTC

*SlPR1* FP: CCGTGCAATTGTGGGTGTC

*SlPR1* RP: GAGTTGCGCCAGACTACTTGAGT

### Statistical analyses

Z-score analyses for the primary quantitative screening was done as per^86^. All statistical analyses done elsewhere were performed in Excel spreadsheets.

## Supplementary information

Supplemental information
