# Supplementary material for: Plant chemical genetics reveals colistin sulphate as a SA and NPR1-independent PR1 inducer functioning via a p38-like kinase pathway
Source: Sci Rep. 2019 Aug 1;9:11196. doi: 10.1038/s41598-019-47526-5 (PMC6671972; doi:10.1038/s41598-019-47526-5)
Supplement: Supplementary file 1 — Supplemental information [file 41598_2019_47526_MOESM1_ESM.pdf]

SUPPLEMENTAL INFORMATION

Plant chemical genetics reveals colistin sulphate as a SA and NPR1-independent *PR1* inducer functioning via a p38-like kinase pathway

Authors: Vivek Halder[*1,2,3], Mohamed N.S Suliman[1,4], Farnusch Kaschani[2], and Markus Kaiser[*2]

[1] Chemical Biology Laboratory, Max-Planck Institute of Plant Breeding Research, Carl-von-Linnè-Weg 10, 50829 Köln, Germany

[2] Chemical Biology, Centre of Medical Biotechnology, Faculty of Biology, University of Duisburg-Essen, Universitätsstr. 2, 45141 Essen, Germany

[3] Present Address: Rijk Zwaan, De Lier 2678 ZG, The Netherlands

[4] Present Address: Desert Research Centre, 11753 El matareya Cairo, Egypt

*__Corresponding authors:__ Vivek Halder (v.halder@rijkzwaan.nl) & Markus Kaiser (markus.kaiser@uni-due.de)

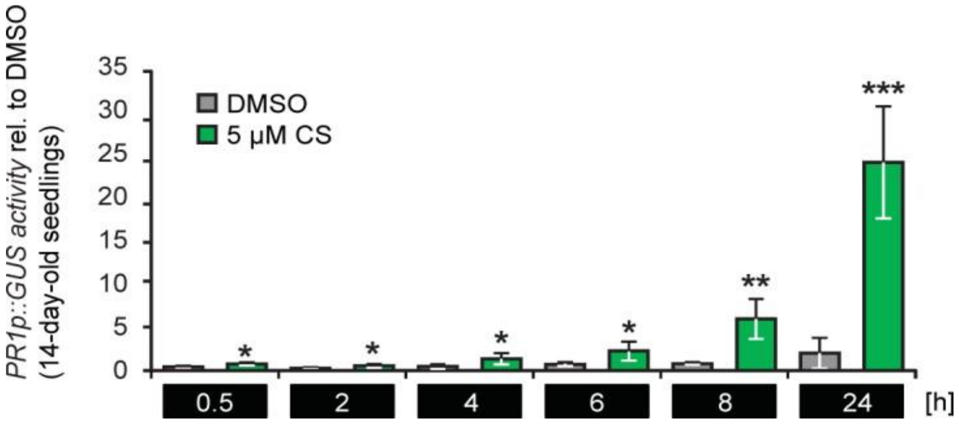

Figure S1: Activation of SA signalling reporter *PR1p::GUS* by CS is time dependent. 14-day-old hydroponically grown *PR1p::GUS* reporter seedlings were treated with **CS** (5 µM) or DMSO (1 %) over a time course of 24 h. Error bars indicate SD. Asterisks indicate significant differences from respective controls (* P < 0.05, ** P < 0.01, and *** P < 0.001, Student's t-test).

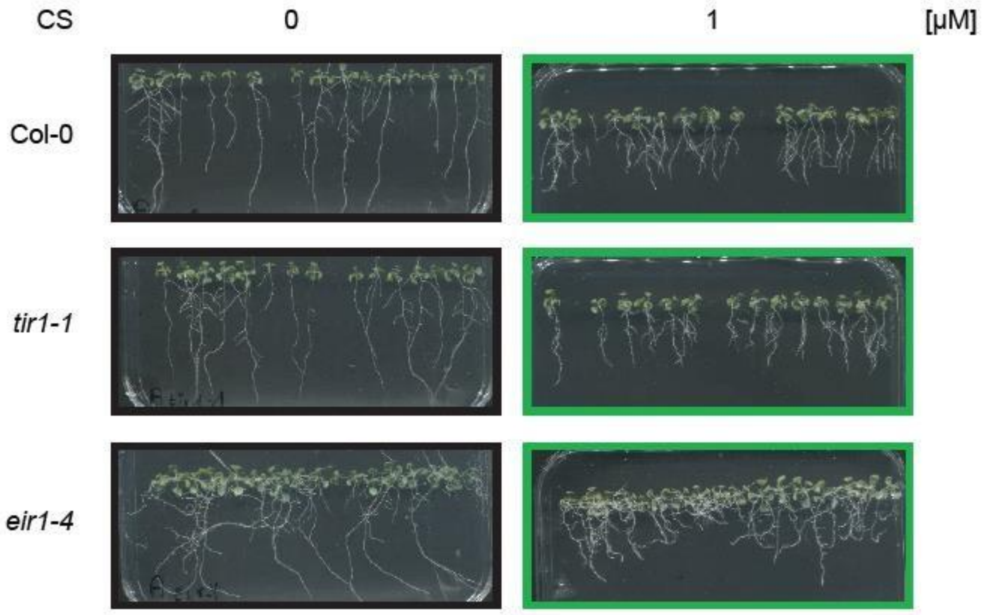

Figure S2: CS' effect on root growth is independent of auxin signalling. Seeds of indicated genotypes were grown on solid half-MS phytagel plates with or without 1 µM **CS** for 12 days under long day conditions.

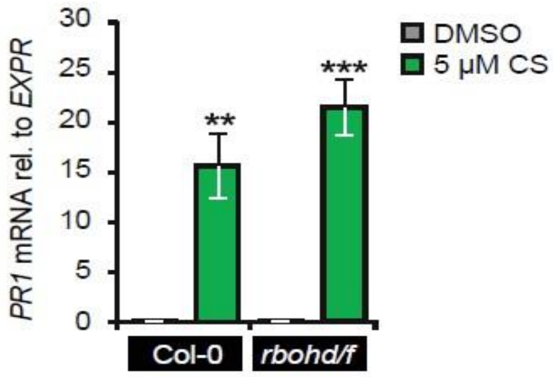

Figure S3: ROS signalling components are not required for CS' bioactivity. 14day-old seedlings of indicated genotypes were treated with **CS** (5 µM) or DMSO (1 %) for 24 h. *PR1* gene expression was quantified via qRT-PCR and normalized to *EXPR*. Error bars indicate S.D. Asterisks indicate significant differences from respective controls (** P < 0.01 and *** P < 0.001, Student's t-test).

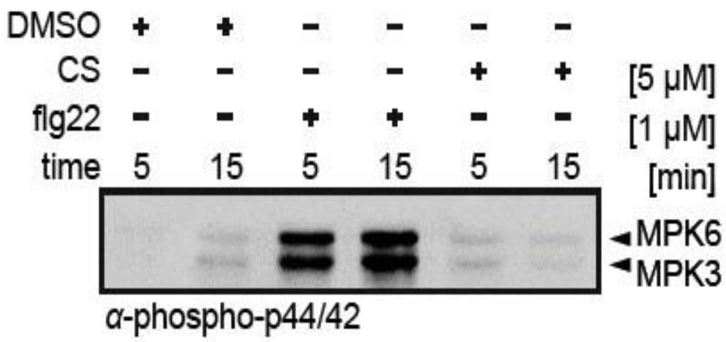

Figure S4: CS does not initiate transient MAPK activation. 10-day-old hydroponically grown Col-0 seedlings were treated with **CS** (5 µM) or DMSO (1%) or flg22 (0.1 µM) for the indicated time points. Total protein was extracted from the frozen plants. Western blot was performed with 10 µg protein in each lane and phosphorylated MPK6 and MPK3 were detected using α-phospho-p44/42 antibodies. For the uncropped blot, see Supplemental **Fig. S9**.

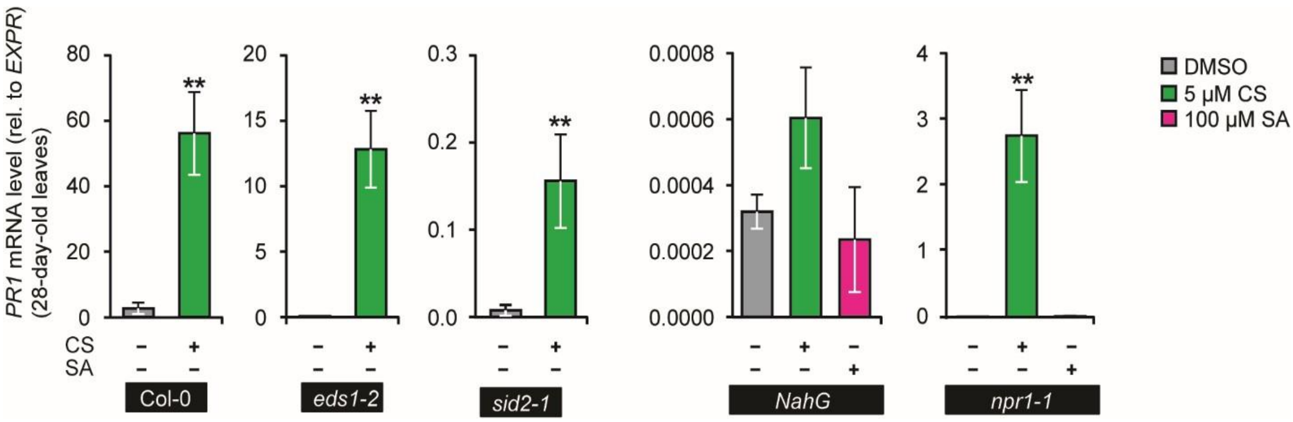

Figure S5: CS' effect is consistent in mature plants. Leaves of different 28-day-old Arabidopsis genotypes were infiltrated with **CS** (5 µM) or SA (100 µM) for 24 h. *PR1* gene expression was quantified via qRT-PCR and normalized to *EXPR*. Error bars indicate S.E.M. Asterisks indicate significant differences from respective controls (** P < 0.01, Student's t-test).

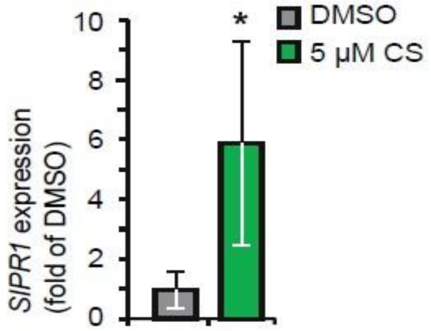

Figure S6: CS induces *PR1* gene expression in tomato. Leaves of four-week-old tomato wild type (*Solanum lycopersicum* cv. Moneymaker) were used to prepare leaf disks. Disks were left in half MS for 1 hour and then treated with the DMSO or **CS** for 24 h. Sl*PR1* gene expression was quantified via qRT-PCR and normalized to DMSO control. Error bars indicate S.D. Asterisks indicate significant differences from respective controls (* $P < 0.1$, Student's t-test).

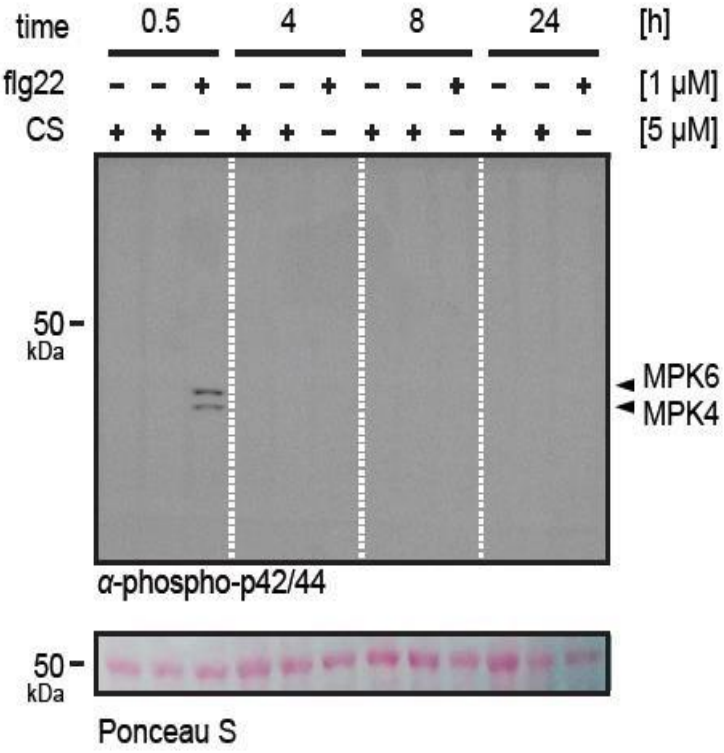

Figure S7: Uncropped Western Blot image of figure 3C.

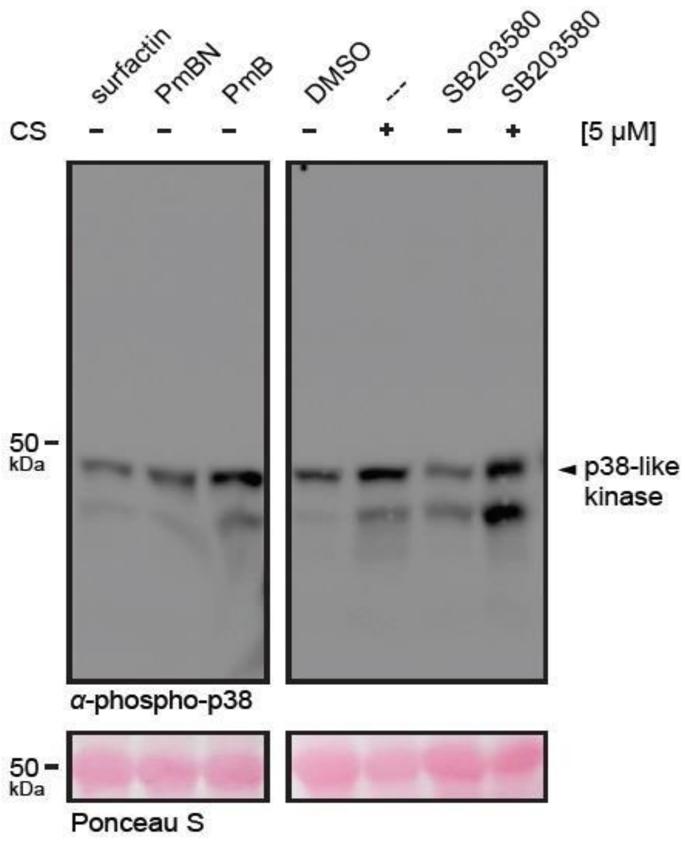

Figure S8: Uncropped Western Blot image of figure 5C.

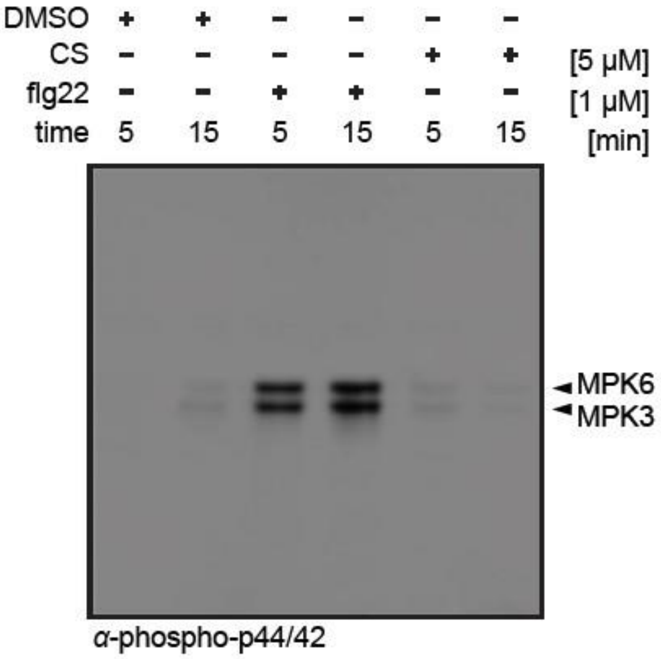

Figure S9: Uncropped Western Blot image of Supplemental figure S4.
